# 3D membrane segmentation and quantification of intact thick cells using cryo soft X-ray transmission microscopy: A pilot study

**DOI:** 10.1371/journal.pone.0174324

**Published:** 2017-04-04

**Authors:** Rubén Cárdenes, Chong Zhang, Oxana Klementieva, Stephan Werner, Peter Guttmann, Christoph Pratsch, Josep Cladera, Bart H. Bijnens

**Affiliations:** 1 Physense, Universitat Pompeu Fabra, Barcelona, Spain; 2 Institute of Neuropathology, IDIBELL-University Hospital Bellvitge, L’Hospitalet de Llobregat, Spain; 3 Experimental Dementia Research Unit, Department of Experimental Medical Science, Lund University, Lund, Sweden; 4 Helmholtz-Zentrum Berlin für Materialien und Energie GmbH, Institute Soft Matters and Functional Materials, Electron Storage Ring BESSY II, Berlin, Germany; 5 Biophysics Unit & Centre of Studies in Biophysics, Dept. of Biochemistry & Molecular Biology, Universitat Autònoma de Barcelona, Bellaterra, Spain; 6 ICREA, Barcelona, Spain; Pennsylvania State Hershey College of Medicine, UNITED STATES

## Abstract

Structural analysis of biological membranes is important for understanding cell and sub-cellular organelle function as well as their interaction with the surrounding environment. Imaging of whole cells in three dimension at high spatial resolution remains a significant challenge, particularly for thick cells. Cryo-transmission soft X-ray microscopy (cryo-TXM) has recently gained popularity to image, in 3D, intact thick cells (∼10*μm*) with details of sub-cellular architecture and organization in near-native state. This paper reports a new tool to segment and quantify structural changes of biological membranes in 3D from cryo-TXM images by tracking an initial 2D contour along the third axis of the microscope, through a multi-scale ridge detection followed by an active contours-based model, with a subsequent refinement along the other two axes. A quantitative metric that assesses the grayscale profiles perpendicular to the membrane surfaces is introduced and shown to be linearly related to the membrane thickness. Our methodology has been validated on synthetic phantoms using realistic microscope properties and structure dimensions, as well as on real cryo-TXM data. Results demonstrate the validity of our algorithms for cryo-TXM data analysis.

## 1 Introduction

Biological membranes provide specialized permeability barriers for cells and cell organelles, in which the interplay of lipids and membrane proteins facilitates basic processes of respiration, photosynthesis, protein and solute transport, signal transduction, and motility [1]. The cell membranes of almost all living organisms and many viruses are made of two layers of lipid molecules (a bilayer) containing various types of protein molecules, which are typically 5–10*nm* in thickness. Nuclei and other sub-cellular structures are also surrounded by one or more lipid bilayers. The composition and content of membrane can be modified in different kinds of cells, including the healthy as well as the pathological ones. The modifications are extensive enough to alter membrane fluidity and affect a number of cellular functions [2]. Such dynamic nature of membranes are also reflected as their structural changes, such as thickening or reduction in membrane thickness, or lipid bilayer width [3–6].

It is very difficult to perform structural studies of membrane because it is fragile and small, much smaller than the diffraction limit of visible light. Traditionally, X-ray scattering techniques were used to calculate membrane thickness from diffraction patterns. In order to see bilayers, fluorescence microscopy with membrane staining was often used, with resolution much smaller than a typical cell but much larger than the thickness of a lipid bilayer. Electron microscopy (EM) offers nanometer resolution similar to the width of a lipid bilayer, but are usually limited to analyzing just a few thin sections from 3D specimens [7]. Cryo-transmission electron microscope (cryo-TEM) based tomography has been used to detect and visualize nanoparticles and membranes [8], as well as some delicate structures which are preserved during vitrification but not in conventional EM fixation [9]. But again, samples exceeding about 500nm in thickness are too thick for imaging and require a thinning sectioning step, which also may produce artefacts in morphology [10]. Such sectioning will also complicate the study of (rare) 3D structures which are more perpendicular to the sections [11]. Cryogenic soft X-ray transmission microscopy (cryo-TXM) is an emerging technique, which is capable of imaging ultrastructure of hydrated intact cells in 3D. The long penetration depth of the X-rays in water, reaching 10*μm*, permits direct observation of intact thick cells in the vitrified state in 3D with a resolution up to several tens of nanometers [12, 13]. The absorption contrast produced by this method can be interpreted quantitatively as a function of material and thickness [14]. In data collection and analysis step, an additional difference between TXM and TEM sectioning or FIBSEM is the speed, which is on the timescale of weeks for seriell TEM sectioning approaches [15]. Therefore, the use of soft X-ray tomography for studying vitrified cells is becoming an important complement to other microscopy methods in observing the structures of organelles [7].

However, detect and quantitatively analyzing membranes on TXM is a challenging task due to the complexity of both the data and the processing procedure. This requires first the identification of membranes in images, also called segmentation, followed by further quantification of membrane thickness, morphology, etc. Currently, this step is often performed manually [8, 16] with the help of software packages like ImageJ, Bsoft, Imaris, Amira, which demands a great amount of time. Much work has been dedicated towards automating segmentation, most of which is targeted to deal with EM data, such as watershed [17], fast marching [18, 19], template matching [20], adaptive shape and points clustering [21], active contours [22], Gaussian-like membrane models [23], ridge-based membrane detector [24, 25] and tensor voting approach [26]. In general, software packages developed for EM tomography are suboptimal when used on TXM data, and there is a need for software dedicated to TXM so as to exploit better these unique characteristics [14, 27]. Segmentation of membranes of thick cells in cryo-TXM data is particularly challenging. First, the membranes are often occluded or altered due to overlap with other neighboring structures. Secondly, the heterogeneous composition as well as background and reconstruction noise, makes the membrane signal not homogeneous along the image. The limited depth of focus is another important issue in cryo-TXM since it only allows to capture details in a limited range along the optical axis (Z axis from now on) of approximately 5 *μm* (for a Fresnel zone plate with outermost zone width of 40 *nm*, as used in this study). A final critical problem is the *missing wedge* effect, produced by the limited tilt range for the projections during our tomographic acquisitions: ±65°. Due to the latter two limitations, the reconstructed volumes are best resolved in XY slices (perpendicular to the Z axis) and located in a limited range of the Z axis [6]. In terms of membrane thickness quantification, an interesting 2D method was presented in [6] to measure organelle membranes, digestive vacuole. The absorption intensity, generated by the membrane in individual 2D tomographic slices that perpendicularly cross it, was translated into the fraction of lipid content for each sampling point, which was interpreted as a local lipid membrane thickness. This resulted in a histogram of the sampling points showing two Gaussian peaks, indicating a single and a double lipid bilayer. However, there has no effective methods yet on 3D intact thick cells.

We propose a methodology to segment and quantify membranes of intact thick cells in 3D using cryo-TXM data sets. Our segmentation method is based on active contours driven by a multi-scale ridge detection. The 3D segmentation is obtained by tracking along the optical axis of the microscope. A quantitative metric, linearly related to the membrane thickness, is then proposed by calculating an area covered by grayscale profiles perpendicular to the membrane surfaces. These profiles are directly related to the absorption coefficient of the organic content [6]. Therefore, the area is directly related to the integrated absorption thus representing the content. We validated both the segmentation and the quantification methods in phantom experiments of synthetic images using realistic microscope properties and structure dimensions. Results show that our tool suggest that the area metric correlates linearly with membrane thickness even for those below the X-ray optical resolution limit. Rather than directly calculating the membrane thickness, our metric is a robust indicator to study native-state membranes. For this, in a pilot application study, we investigated the interaction between biological membranes in human neuroblastoma cells, illustrating how the techniques proposed can provide quantitative membrane measures on real data sets.

## 2 Materials and methods

### 2.1 3D Membrane segmentation

**Fig 1:**
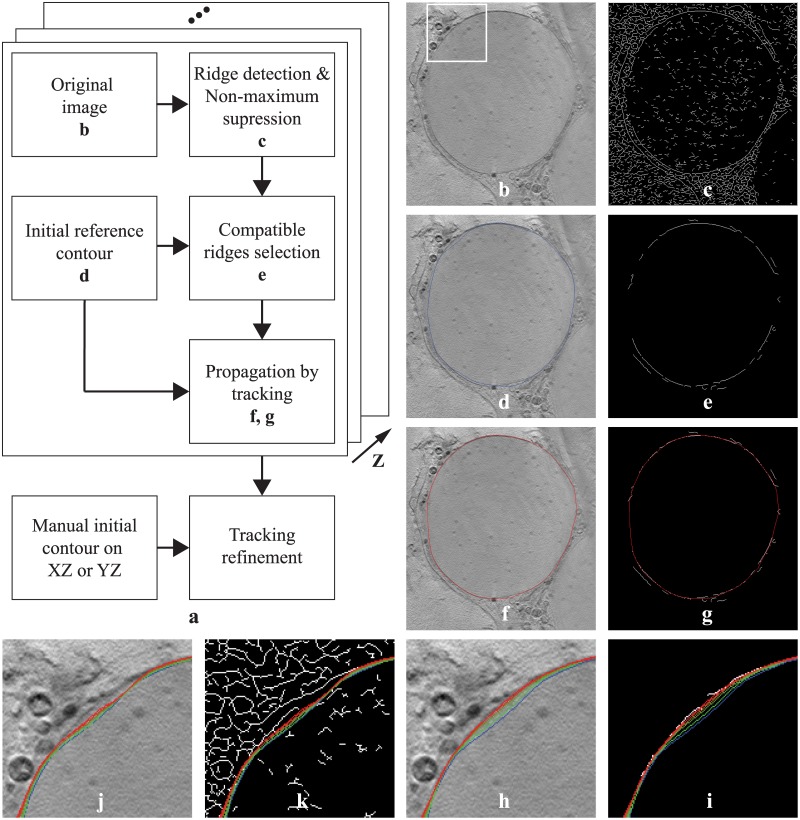
(**a**) Schematic diagram of the segmentation algorithm. To illustrate better the algorithm procedure steps, intermediate results from an example image of the real data (described in Section 2.5) are also demonstrated: starting from the original image (**b**), a local multiscale ridge detection step is first applied (**c**), followed by a ridge selection or pruning step (e) to keep only those compatible with an initial (manual) contour (**d**, *blue*) from a reference slice, the latter is then deformed to detect the current membrane contour (**f, g**, *red*) through an active contour based model that is driven by the found compatible ridges. 3D membrane segmentation is obtained via propagation along the optical axis (Z axis). Additionally, an optional refinement step along the YZ and XZ slices is proposed to cope with cryo-TXM imaging limitations. Inset view (*white frame* in a) with the contours sequence obtained from the segmentation process in 5 iterations, from initial guess (*blue*) to the solution (*red*), driven by compatible ridges only (**h, i**), or by all detected ridges (**j, k**).

Our segmentation approach comprises of two steps: firstly, a local ridge detection and selection procedure is performed to find “compatible ridges” on each 2D slice (cross section), oriented similarly to an initial contour; secondly, an active contour based model is initialized and deformed from a particular slice to propagate along the axis perpendicular to the slice, driven by the found compatible ridges. Since cross sections do not change abruptly throughout most of the cell, such 3D segmentation through 2D detection and propagation through tracking approach, which is also adopted in [21, 22], is effective and keeps computational complexity manageable even on large data sets. Additionally, in order to cope with the limited focus depth and the missing wedge effect, that are especially pronounced in XZ and YZ planes for thick cells, an optional refinement step along the YZ and XZ slices is proposed to recover the entire object. A schematic diagram of the algorithm is shown in Fig 1a.

#### 2.1.1 Ridge detection and selection

Since membranes appear as elongated dark structures, a multi-scale ridge detection with automatic scale selection (introduced in [24]) is used. This method is suitable since it provides the compatible ridges of the image that correspond to parts of real organelle membranes, and it is robust with respect to the scale. We briefly summarize how the ridges are obtained. The ridges of an image are defined as the local maxima over a range of scales of a ridge measure defined in the scale space representation of the original image *I*:

$$\begin{array}{c} L(x,t)=g(x,t)*I(x), \end{array}$$ (1)

where *g* denotes a Gaussian kernel, and *t* the scale:

$$\begin{array}{c} g\left(x,t\right)=\frac{1}{2\pi t}e^{-\left(x^{2}+y^{2}\right)/\left(2t\right)}. \end{array}$$

With this scale space representation, let M:L↦R be a measure of ridge strength, which is a *γ*-normalized maximum absolute eigenvalue of the Hessian matrix [24] of *L*:

$$\begin{array}{c} M(L)=t^{\gamma }max(|L_{pp}\left|,\right|L_{qq}\left|\right), \end{array}$$

where *γ* is a normalization factor, and *L*_*pp*_ and *L*_*qq*_ are the eigenvalues of the Hessian matrix, which are considered as the main curvatures at each image pixel location. The ridges of the image *R* are defined as those having locally maximal ridge strength with respect to scale, argmax*_L_ M*(*L*).

However, due to noise, reconstruction artifacts and overlapping structures, some detected ridges do not belong to a real membrane. They are partially removed in the following steps. First, the ridges detected are pruned using a non-maxima suppression:

$$\begin{array}{c} R_{m}\left(x\right)=\left\{\begin{array}{cc} R(x) & R(x)>R(\pm \nu (x))\\ 0 & \text{otherwise} \end{array}\right., \end{array}$$

where *ν*(***x***) is the unit vector in the perpendicular direction to the ridge at pixel *x*. Then, an Otsu threshold is performed to obtain a binary image containing a collection of ridges that represent candidates of parts of membranes in the image: $R_{b}=\cup_{j=1}^{M}\Gamma_{j}$, where each individual ridge, or arc segment, is a connected set of pixels between two end pixels, two branching pixels or between an end pixel and a branching pixel.

To further prune the ridges from {Γ_*j*_} in each image *I*_*i*_, an initial closed reference contour, *S*_*r*_, is first manually selected at one particular XY slice of the volume *V* (*x*, *y*, *z* = *r*) = *I*_*r*_, by selecting *N* membrane points: $S_{r}=\cup_{i=1}^{N}\sigma_{i}=\cup_{i=1}^{N}\hat{p_{i}p_{i+1}}$, with ***p***_*N*+1_ = ***p***_1_. Next, only the ridges that are locally compatible with *S*_*r*_ are preserved. Compatible ridges *R*_*c*_ are large enough segments that at the same time have the same orientation in a local neighborhood of *S*_*r*_:

$$\begin{array}{ll} R_{c}= & \{\Gamma_{k}|\Gamma_{k}\subset R_{b}\}\text{,}\\ \text{s}.\text{t}. & d\left(\sigma_{i},\Gamma_{k}\right)<\rho \;\exists i\in \left\{1\ldots N\right\}\text{;}\\ & |O\left(\Gamma_{k}\right)-O\left(\sigma_{i}\right)|<\theta \;\exists i\in \left\{1\ldots N\right\}\text{;}\\ & |\Gamma_{k}|\geq \varepsilon \forall k\text{,} \end{array}$$ (2)

where *d*(*σ*, Γ) is the average distance from *σ* to Γ, and *O*(Γ) is the average orientation of one arc segment. Segments smaller than *ε* are excluded in order to avoid including nearby segments originating from noise.

#### 2.1.2 Propagation through tracking

We model the 3D surface as a set of *C* contours, {*S*_*k*_ ∣ *k* ∈ {1…*C*}}. Each contour *S*_*k*_ is obtained by propagating points on the initial reference contour *S*_*r*_ towards the membrane contour on image slice *I*_*k*_, guided by an energy model. By tracking the contours forward and backward along the Z optical axis, we can find all subsequent contours, comprising the 3D segmentation of the membrane. Our model is based on active contours or snakes [28], making use of the found compatible ridges to drive the snake deformation:

$$\begin{array}{c} E_{snake}=\sum_{i=1}^{N}\;E_{int}\left(p_{i}\right)+E_{ext}\left(p_{i}\right) \end{array}$$ (3)

where *E*_*int*_ restricts the contour to be smooth and *E*_*ext*_ forces the contour to move towards the ridges in the image *I*_*k*_. The internal energy term *E*_*int*_ is expressed similarly as in [22]:

$$\begin{array}{c} E_{int}\left(p_{i}\right)=\frac{1}{2}\;\left(\alpha ||p_{i}-p_{i-1}{\left|\right|}^{2}+\beta ||p_{i-1}-2p_{i}+p_{i+1}{\left|\right|}^{2}\right), \end{array}$$ (4)

where the first term prevents large gaps in the contour and the second one forces the contour to be smooth. *α* and *β* are weight parameters. We introduce a function of the compatible ridges *R*_*c*_ in Eq 2 as the external energy term:

$$\begin{array}{c} E_{ext}=\kappa \;D\left(R_{c},p_{i}\right), \end{array}$$ (5)

where *D*(*R*_*c*_, ***p***_*i*_) is the Euclidean distance between the contour point ***p***_*i*_ and the closest compatible ridges and *κ* balances the contribution between the external energy and the internal energy. By substituting Eqs 4 and 5 into Eq 3, we obtain the energy to be minimized iteratively. We use the same minimization procedure as in [28].

#### 2.1.3 Tracking refinement

Due to the limitations of the focus depth and missing wedge effect, there is a substantial loss of signal outside the focused and low tilt angle part of the volume, causing the tracking procedure to be unreliable in these regions. However, when looking at the YZ and XZ planes, the entire object shape can still be identified. Therefore, to correct the segmentation when a small portion of the object is affected by these problems, an optional refinement step along the YZ and XZ slices is also proposed to recover the entire object. This step is also a tracking procedure that starts from a manually defined contour at slice *V* (*x* = *m*, *y*, *z*) or *V* (*x*, *y* = *l*, *z*). The contour propagation in this case uses the segmentation, obtained previously in the tracking along Z, as the compatible ridges to control the snake deformation.

### 2.2 Simulated cryo-TXM tomogram

**Fig 2:**
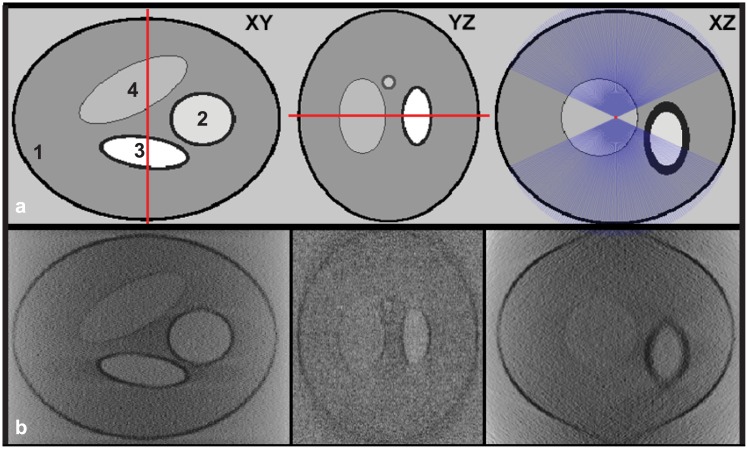
(**a**) Digital phantom simulating 4 organelles. The tilt axis is marked in *red* and the tilting angles (±65°) for the projections are marked in *purple lines*; (**b**) reconstruction from simulated X-ray microscope data.

To validate the segmentation algorithm, we simulated a synthetic phantom mimicking: the characteristics of cryo-TXM imaging, including the missing wedge effect, blurring and noise of the optics, the digital image resolution and the structural size of a thick cell with plasma and other organelle membranes. In particular, we modified a Shepp-Logan digital phantom [29] to represent a simplified geometry of cells. Four different models were generated, simulating four organelles (Fig 2). It was first blurred with a gaussian kernel to make the sharp boundary transitions more realistic. To simulate the missing wedge effect, 131 projections in the range of ±65°, with a 1° step (Fig 2a), were generated using a tilt axis (indicated by the red lines in Fig 2a) parallel to the Y axis and passing trough the volume center, with the 0° tilt projection perpendicular to the Z axis. Then, each projection was convolved with a gaussian kernel to approximate the X-ray microscope image formation with a point spread function (PSF) in ideal situations [27, 30]. Finally, the 3D reconstruction was done with the filtered back-projection algorithm in IMOD [31]. The reconstructed volume is shown in Fig 2b, where noise and artifacts caused by the missing wedge effect are clearly visible, especially along the Z axis and away from the tilt axis, i.e. defocused regions.

### 2.3 Membrane quantification

Once the membranes are reliably segmented, they can be further analyzed and quantified. However, we do not intend to measure absolute membrane thickness, but rather seek for a measure that is directly related to it, linearly, if possible. This is because: 1) It is expected that the membrane thickness is not directly resolved using TXM. Nonetheless, given the existence of a direct relationship between the X-ray absorption and image gray-scale value [14], useful contrast may be generated by smaller structures [5, 6]; 2) Quantification through the seemingly intuitive absorption contrast poses several uncertainties for obtaining absolute values of the absorption coefficients of the membranes. For example, the unknown thickness of the vitreous ice layer covering the sample results in different absorption for similar concentration of organic material. Also, materials with known absorption coefficient values may be absent in the image, making the exact calibration of the image gray-scale value almost impossible; 3) In practice, most applications are interested in looking for structural changes in membranes, thus it would be sufficient to be able to detect relative changes in a comparative way.

We propose a quantification approach based on the analysis of the variation of the image grayscale values along points perpendicular to the membrane 3D surface, i.e. the membrane surface normal direction. To do that, we construct what we denote as membrane profiles, which provide information related to the absorption properties of the membrane and its surroundings [6]. Therefore, the area covered by the profile is directly related to the integrated absorption, thus representing the content. For each surface point, a profile is obtained storing the volume values along a line traced on the membrane normal, from the inner to the outer direction. Since individual profiles are noisy and do not have a direct meaning to explain properties on organelle level, membrane profiles are calculated by the average of those at every point in local regions. The reasons that we have calculated the profile on local regions rather than on the whole cell are: first, we could not assume that there is homogeneous membrane structure everywhere on cells as well as its structural changes when occur; second, we do hope to analyze local structural change distribution along the whole cell surface.

**Fig 3:**
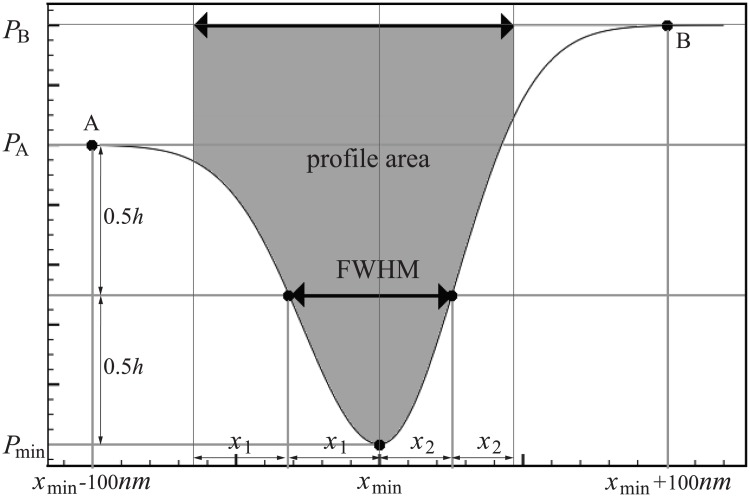
Schematic drawing of the profile area (grey region) based on membrane profile.

A graphical illustration of how the profile area is defined is shown in Fig 3. To calculate the profile area, an adapted full width half maximum (FWHM) of the profile is first defined: the maximum height is set as the lower of the two reference points (*A* and *B* in Fig 3) at a certain distance from the profile peak (*P*_*min*_ in Fig 3). Here the distance is set constantly as ±100*nm*, enough to cover the amount of membrane-related absorption spread in the image due to the microscope optics. The profile area is then computed from the region enclosed by the profile, the line of the higher profile height, and the two lines located at a distance away from the profile peak at both sides, i.e. double the width of the FWHM of the profile (*x*_*min*_−2*x*_1_ and *x*_*min*_+2*x*_2_ in Fig 3).

### 2.4 Simulated membrane model data sets

To evaluate the proposed membrane profile quantification from images produced by an X-ray microscope, we demonstrate, through synthesized phantoms (Section 3.2.1), how it scales with respect to the theoretical membrane thickness. First, curvy contour models are created to simulate a shell-like membrane, next simulated images are generated to mimic the characteristics of data produced by such an X-ray microscope.

A single shell as well as a double shell model are simulated so as to mimic organelles with either single bilayers or double bilayers (such as the nuclear envelope). Particularly, the single shell model has a theoretical thickness *δ*, and the double shell model consists of two concentric annuli, each of which also has a theoretical thickness *δ*, leaving a space between them of the same width. Due to the dynamic nature of membranes, for example, when lipid multilayer patches of extra thickness could be expected in some membranes [5], or the reduction in the lipid bilayer width of some pathology cells [3, 4], the theoretical thickness was parameterized from 5*nm*, corresponding to a single lipid bilayer, and up to 50*nm*, so as to make sure that the considered or observed variation range in membrane thickness is included. Specifically, 10 phantoms of the single shell model (5–50*nm*) and 8 phantoms of the double shell model (5–30*nm* each shell) were used.

**Fig 4:**
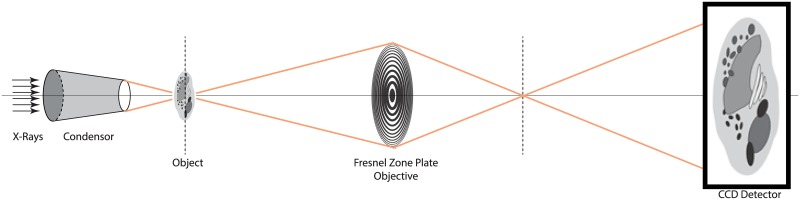
Schematic drawing of the cryo-TXM optical settings.

**Fig 5:**
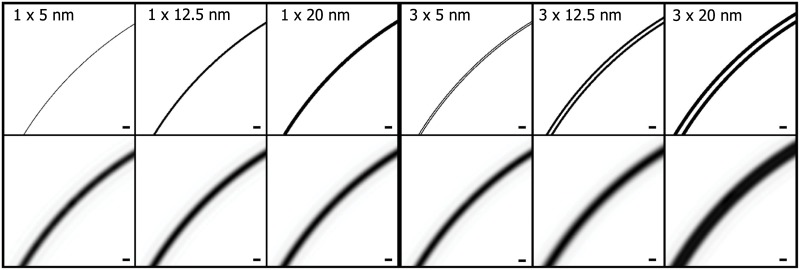
(*Top row*) Examples of the two contour models: single shells (e.g. to mimic plasma membranes) of various thickness values (three *leftmost*) and double shells (e.g. to mimic nuclear membranes) of various thickness values as well as with the space between the two bilayers (three *rightmost*). (*Bottom row)* The corresponding images after convoluted with the PSF of the X-ray microscope at the TXM-U41 beamline of BESSY-II. The scale bar equals 40*nm*.

Assuming the specimen is in focus, the image *I*_*im*_ obtained at the detector can be expressed as the convolution of the PSF of the microscope with the intensity in the object plane *I*_*object*_ [30], which is the contour model in our case. A schematic drawing of the imaging system is shown in Fig 4. The intensity contrast of *I*_*object*_ is simulated as a function of membrane thickness and absorption coefficient. Ideally, *I*_*object*_ has pixels of size “infinitely” small. In practice, it is enough to be discretized with a sampling size equal or smaller than half of the finest detail to be observed. In our case, we set it to Δ*x* = 2.3 nm, around half of the membrane thickness. That means membrane pixel values are set to *e*^−*μ*_*m*_ Δ*x*^, where *μ*_*m*_ is an estimation of the absorption coefficient of the membrane, taken as *μ*_*m*_ = 1.03 *μm*^−1^ (that of the constituent lipid molecules) [6]. Since, in this study, we are only interested in membranes and the measures on local image grayscale profiles around membrane pixels, for simplicity, we assume the membrane is locally surrounded by one type of content composition, either with purely one material content or a known ratio of mix, with known absorption coefficient(s). In this case, we consider the surrounding content is vitreous ice, thus the rest of the pixels are set to *e*^−*μ*_*ice*_ Δ*x*^ using the absorption coefficient of vitreous ice *μ*_*ice*_ = 0.109 *μm*^−1^. Although image formation in a real X-ray microscope is complex, a constant PSF of a perfect system has been shown to be reasonable in X-ray Tomography, even for thick specimens [27]. Therefore, the PSF is approximated by the image produced by the Fresnel Zone Plate (FZP) from a point source on the optical axis. Here, it is computed as follows: where the FZP is used is discretized on a rectangular grid and the propagation of the incident light is computed by the convolution of the Fresnel diffraction as in [32], using a Fresnel zone plate with outermost zone width of 40*nm*. Examples of the two membrane models *I*_*object*_ are shown in Fig 5 *top*, and their corresponding images *I*_*im*_ after convolution with the PSF can be found in Fig 5 *bottom*.

### 2.5 Real data sets

To show the feasibility and performance of our membrane segmentation and quantification methodology, we have applied it to data from whole, unsliced, thick cells imaged with a cryo-TXM. The experiments were carried out following the guidelines of the ethics committee of University of Barcelona.

Specifically, these data were part of a study to investigate the structure and dynamics of Amyloid *β*-peptide and its interaction with biological membranes of SH-SY5Y human neuroblastoma cells exposed to it. A*β* peptide is a toxic found in brains of AD patients, and although its precise role in AD remains inconclusive, the current consensus is that it is a key player in the development of the disease. One hypothesis on how A*β* leads to neuronal degeneration and toxicity states that the hydrophobicity of extracellular A*β* disrupts the bilayer permeability of neuronal membranes [33]. Therefore, the analysis of membranes in these cells is of particular interest.

The typical sample preparation for cryo-TXM acquisition requires growing cells directly on microscopy grids, i.e. Au HZB-2 grids (Gilders Grids, UK), coated with Formvar and Carbon. These cells have flat areas up to 30*μm*^2^ with a thickness between 3–5*μm*. A monolayer of cells, grown on the grid, was created under controlled conditions. A set of cells treated with A*β*(1-40) peptide, according to the protocol described in [34], were also included. To facilitate subsequent image alignment and 3D reconstruction, 250*nm* colloidal gold particles (BBInternational, UK) were added to serve as fiducial markers. Afterwards, to prevent water from turning into crystalline ice and to maintain the sub-cellular structures intact, cells were fast frozen by plunging [12] in liquid ethane, using a Cryoplunge^™^3 (Gatan, Germany). The frozen grids were imaged at a synchrotron soft X-ray microscope (beamline U41 XM at the electron storage ring BESSY II, Berlin, Germany) with a photon energy within the water window (510 eV), to take advantage of the high natural contrast of biological material compared to water, according to the conditions described in [12, 35]. The cryo-fixed sample was rotated between ±50° and ±65° degrees with steps of 1°. The series were collected using a zone plate objective with an outermost zone width of 40*nm* to maintain the cell cytoplasm in focus, resulting in a spatial resolution of the tomographic reconstruction of about 60 nm (full period). With these settings, we obtained image projections of dimension 1324 × 1284 with effective pixel sizes of 15 nm. The image stacks were pre-processed to normalize and correct the intensity distribution delivered to the sample by the capillary condenser using flat field images. The projections were aligned by cross-correlation and manually selected fiducial markers using eTomo from the IMOD software package [36]. Regions of major interest, i.e. plasma and nuclear membranes with surroundings, were reconstructed in 3D using the filtered back-projection method in IMOD.

## 3 Results and discussion

In order to evaluate the performance of our methodology of segmentation and subsequent membrane quantification, the above described experiments have been carried out. The first set of experiments uses the phantom made from simplified geometries similar to biological structures with realistic properties of a cryo-TXM system, so as to validate our segmentation algorithm. The second set of experiments uses the phantom mimicking biological membranes with variations, in order to validate our membrane quantification metric. And the last set of experiments uses the measured data obtained from a synchrotron soft X-ray microscope, to investigate the feasibility of applying our method to real biological problems.

### 3.1 Phantom experiments

#### 3.1.1 Validation of the segmentation method

**Fig 6:**
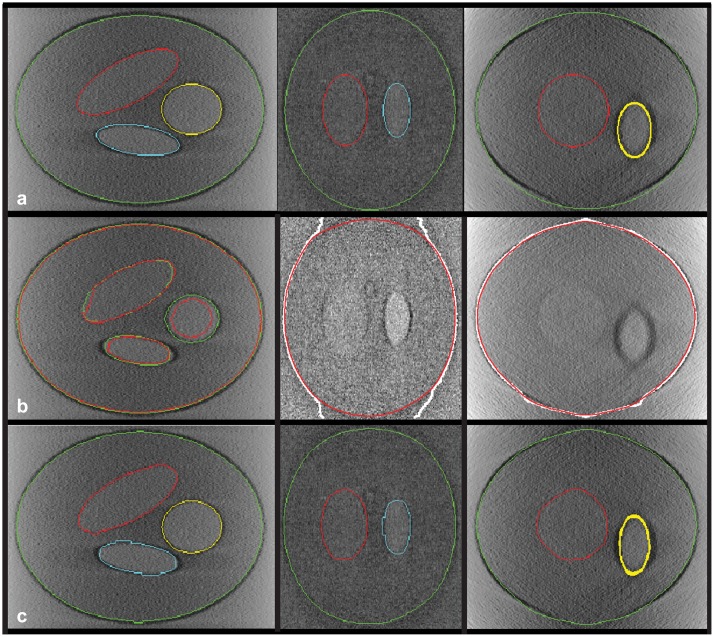
(a) Reconstruction from simulated X-ray microscope data and overlaid with known contours from the ideal geometry; (b) *left*: XY slice overlaid with the segmentation, using the found compatible ridges only (*green*) or using all ridges (*red*); *middle* and *right*: YZ and XZ slices overlaid with segmentation results before (*white*) and after (*red*) the refinement step for the membrane of organelle 1; (c) reconstruction overlaid with the final segmentation.

The segmentation with our method is shown in Fig 6. It was segmented along the Z axis, followed by two refinement step along the X and Y axes. The refinement steps have helped improving the segmentation to handle the problems of the limited focus depth and the missing wedge effect. An example of Organelle 1 is shown in Fig 6b, demonstrating how the white contour obtained by tracking along the Z axis has been corrected (shown as red contours) by the refinement steps.

**Fig 7:**
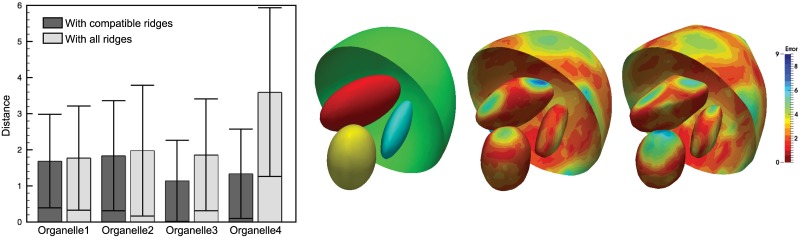
*left*: Grouped bar plot of average segmentation error and standard deviation, calculated as surface distance, from the results with compatible ridges only and with all ridges; *right*: ideal geometry of the phantom, Color-coded segmentation errors from the results with compatible ridges only and with all ridges.

To evaluate the usefulness of the compatible ridges selection, a baseline method is used by modifying the external energy term in Eq 5, instead of the compatible ridges *R*_*c*_, all the detected ridges *R*_*b*_ are used. Results comparing them are shown in Fig 2d before applying the refinement steps, and after applying them (Fig 7), with a bar plot of the distances (in pixel units) obtained for each 3D segmentation surface with respect to the ideal geometry. Segmentation results using all the ridges show slightly higher errors than those using the compatible ridges only. Qualitative comparison of the results is also shown in Fig 2d for one slice and in Fig 7 as 3D surface errors, where each segmented model is color-coded using the nearest distance to its corresponding reference model.

#### 3.1.2 Validation of the membrane profile analysis

**Fig 8:**
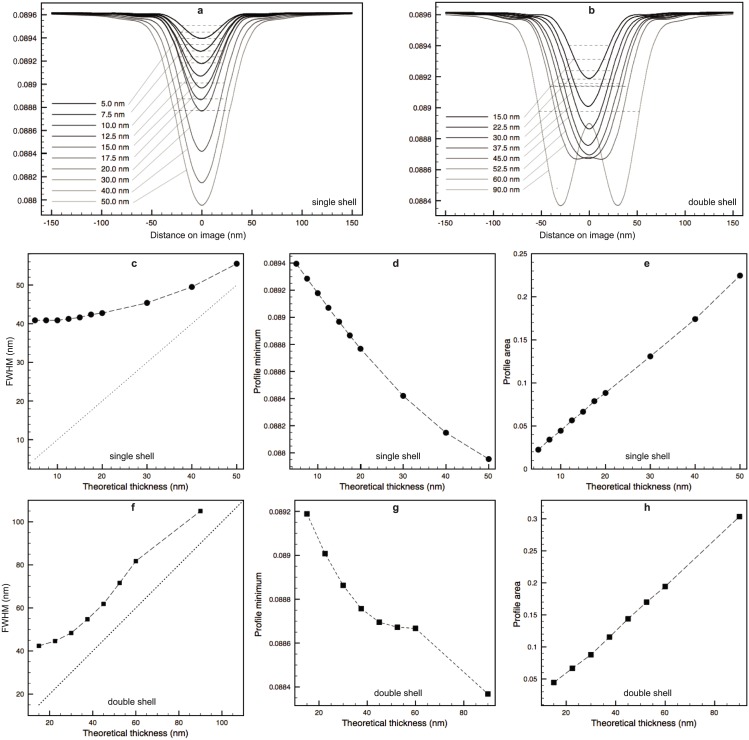
Average membrane profiles with variable thickness obtained for single shell (**a**) and double shell (**b**) phantoms. Plots of three different features of the profiles against the theoretical thickness for both models: FWHM (**c, f**), profile minima (**d, g**) and upper area (**e, h**).

The regions analyzed with membrane profiles are confined to be within the central section that is least affected by the missing wedges and limited focus depth. The average profiles are computed for each phantom and shown in Fig 8a and 8b for single shell phantoms and double shell ones, respectively. The double shell profile peaks are detectable when *δ* ≥ 20*nm* (and a total thickness of 60*nm* or higher) amongst the tested phantom cases. However, although for those where the double shell is not resolved, the profiles vary in both contrast and width, i.e. larger contrasts and wider peaks as the thickness increases. We have measured three different features of the profiles with respect to the membrane thickness: the profile FWHM, the profile minima and the profile area (defined in Section 2.3 and Fig 3). The obtained FWHM for all the phantoms, are marked in Fig 8a and 8b (dashed lines). Fig 8c–8h plots these three measures against the theoretical shell thickness for both models. As expected, the shell thickness values can not be inferred directly using the typically represented FWHM of the membrane profile measured on images, because their sizes are much smaller than the optical resolution of the microscope. Also, its relation with the thickness is different for the two models, as can be observed from the curves shown in Fig 8c and 8f. This would impose an ambiguity of the choice of the model when it is unknown if the membrane examined is a single or double bilayer, e.g. in pathological cells. The same ambiguity holds also for the profile minima, as shown in Fig 8d and 8g. Whereas in the case of the proposed area metric (Fig 8e and 8h), both the single and double shell models exhibit a nice straight line in the plots, suggesting a linear relationship between the area metric and the theoretical thickness of the shell, even for a thickness below the microscope resolution.

**Fig 9:**
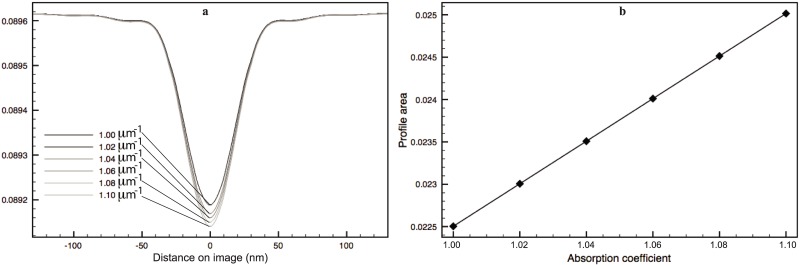
Average profiles for the phantom with thickness 15 nm, with variable *μ*_*m*_. G: Upper profile area with variable *μ*.

Whether changes in the concentration or composition of material content can be reflected in the area metric is also investigated. Profiles are computed using different absorption coefficients *μ*_*m*_, varying slightly from that of the lipid, so as to mimic different concentration of lipid in the membrane. Indeed, different profiles suggest that these small changes in absorption coefficient could be captured. Again, a linear relationship is observed between the area metric and the absorption coefficient. As an example, Fig 9a plots the profiles computed on the single shell model with 15*nm* shell thickness. The area values obtained for these profiles are also plotted in Fig 9b.

### 3.2 Real data experiments

#### 3.2.1 Segmentation

**Fig 10:**
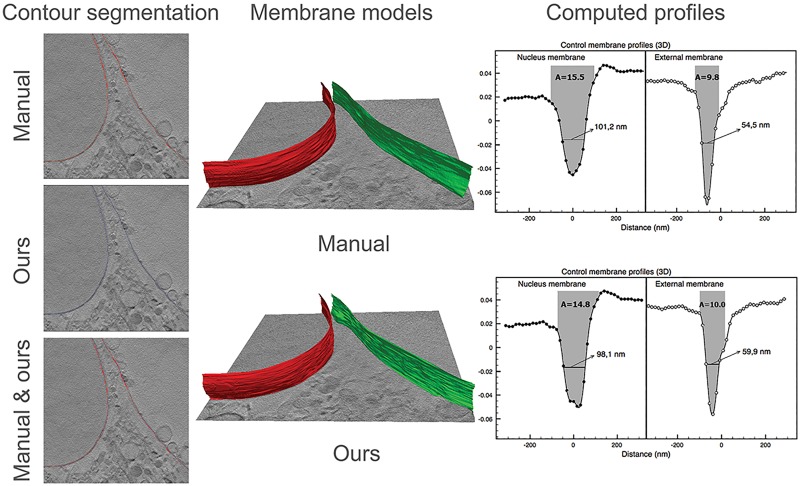
A segmentation example for a local region in a control cell, using both the manual segmentation and our algorithm. *left*: Segmentation contours overlaid on a 2D slice; *middle*: The 3D surface models obtained from both segmentations; *right* And the average plasma and nuclear membrane profiles, as well as their FWHM and upper area values.

Since the ground truth of the membrane location and profile is unknown, in Fig 10, an example of the manual segmentation and our algorithm for a control cell is shown, as well as subsequent profile measures calculated on both segmentation results. Our segmentation is comparable to the manual delineation on the entire measured local region, thus resulting in also similar profile area measures.

**Fig 11:**
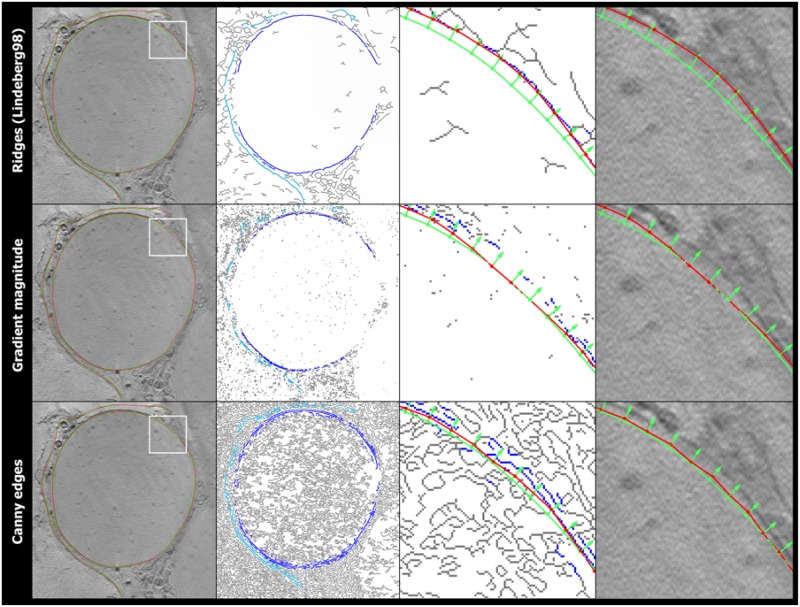
Results of different boundary detection algorithms to extract compatible edges on slice of a cell (from *top* to *bottom*): multi-scale ridge detection [24] we used, gradient magnitude, and Canny edge detection. (from *left* to *right*): Obtained contour (*red curve*) overlaid on the slice image, together with the initial contour (*green curve*), and a white frame indicating the zoomed in views on the next columns; All detected edges (*grey curves*) and those compatible ones (*blue curves*); Zoomed view of all detected edges (*grey curves*), compatible ones (*blue curves*), the initial contour (*green curve*) with its normal direction (*green arrows*), and the obtained contour (*red curve*); Same zoomed view with contours overlaid on the slice image. Same initial contour was used for all results from different boundary detection algorithms.

We propose to segment the cells based on membrane boundaries because of their distinguishable contrast with surroundings. However, one common challenge is that features extracted from cryo-TXM images (as well as EM images [21]) are represented only as discrete boundaries rather than a closed form of surface or contour. Also, regular edge detection algorithms would detect many false positive edge fragments as membrane of interest, therefore any edge detection method alone would not work. Therefore, in order to have a meaningful comparison, we have applied Canny edge detector and gradient magnitude to obtain “edges”, and then employ the same steps of our proposed compatible edges selection and contour propagation on these differently detected “edges” to obtain the membrane segmentation. Fig 11 shows an example results based on these different membrane boundary detection algorithms. Our method could find the correct contour, while the other two methods could not find correct “edges” to guide the propagation of initial contour, resulting in wrongly segmented membrane doundaries. Furthermore, membrane segmentation becomes very challenging when it is to serve for membrane quantification later, e.g. to find an accurate contour matching the membrane center, when taking into account noise, artifacts, membrane gaps, and touching organelles. Of course other approaches based on machine learning could also be used, however machine learning is subject to a selection of enough training data which would be cumbersome in situations with low number of data as could be TXM cases.

The parameters used for the membrane segmentation of our experiments are summarized in Table 1. Specifically, we set: in Eq 1 the scales *t* ∈ {10, 15} for ridge detection; In Eq 2, *ρ* = 70*nm*, *θ* = 30° and *ε* = 50*nm*. This means that each point on the initial contour is displaced a maximum of 70*nm*, and the detected ridge segments whose orientation differ more than 30° with respect to the initial contour segment in this region are disregarded; In Eq 4, *α* = 0.01 and *β* = 0.01; in Eq 5, *κ* = 8); The number of iterations was set to 5 since our model converges fast and the initial contour was usually close to the membranes in other slices. Regarding parameters selection, some parameters are not data specific, e.g. *α* and *β* only affects smoothness of the segmented contour, while other parameters should be changed only if the target structure, resolution or any other important image data characteristic is changed. Therefore, although they were set empirically for our experiments, we expect that these values are good enough to be used as default values for segmenting thick cells with diameter of a couple of micrometers.

**Table 1:** Parameters used for membrane segmentation.

Method	Parameter usage	Value
Ridge detection in Eq 1	scale *t*: use more scales if inhomogeneous membrane boundaries; use larger values if image is noisy	{10, 15}
Ridge selection in Eq 2	*ρ*: set larger if allow initial contour is farther *θ*: set larger if cell/organelle is smaller *ε*: set larger to include less but longer ridge segments	70*nm* 30° 50*nm*
Internal energy in Eq 4	*α*: set larger to penalize too few contour points *β*: set larger to get smoother contour	0.01 0.1
External energy in Eq 5	*κ*: set larger if initial contour is not far from true ones # iteration	8 5

#### 3.2.2 Cell membrane quantification

**Fig 12:**
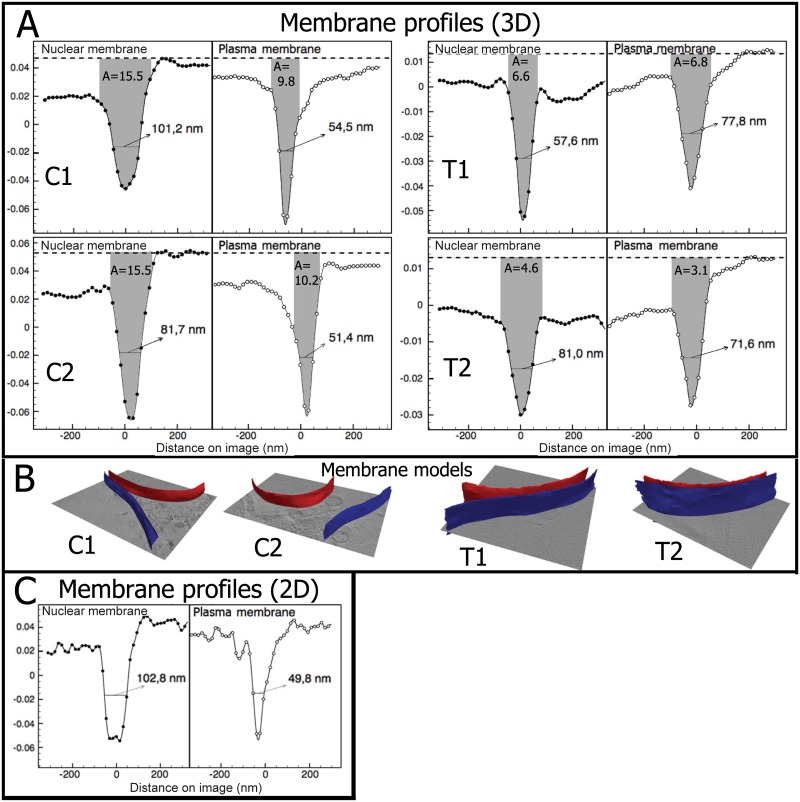
A: Membrane average profiles computed in local regions from 2 control and 2 incubated cells. Negative values indicate interior of the nucleus/cell; B: membrane surface models used for the 3D profiles computation (blue: plasma membranes; red: nuclear membranes); C: membrane average profiles computed in 2D for a single slice of C1.

**Fig 13:**
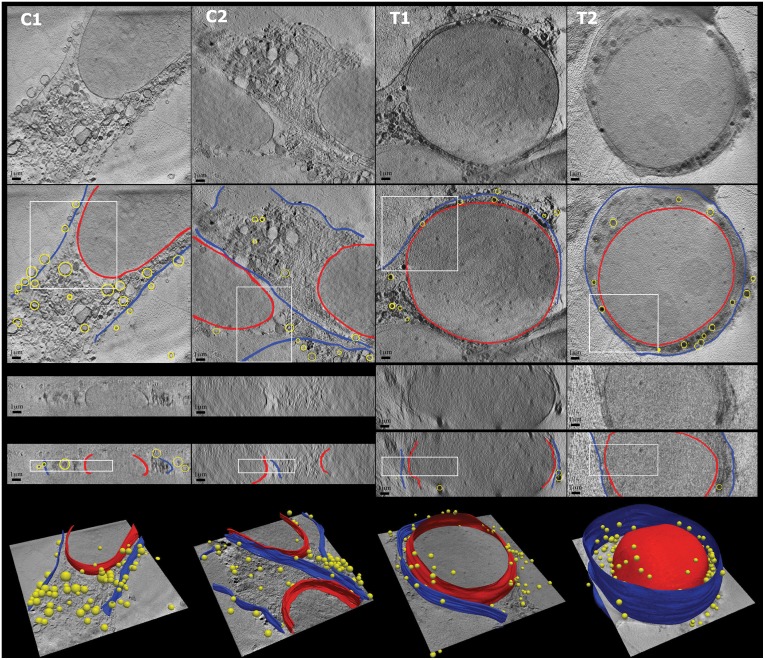
*Top rows*: Examples of control cells (C1, C2) and treated cells (T1, T2) of the original reconstruction and the segmentation as contour overlay in XY and XZ views: plasma membrane (*blue*), nuclear membrane (*red*) and other organelles (*yellow*). Rectangles indicate the local regions used for the plasma and nuclear membrane profile analysis shown in Fig 12. *Bottom row*: 3D surface view of the segmentation. The circular sub-cellular organelles (shown as yellow spheres) were segmented using a variant of the Circular Hough Transform [37].

For the membrane quantification, regions where the missing wedge effect and the out of focus effect were minimum in the reconstruction are selected. That means, measured membrane parts are those that approximately cross XY slices perpendicularly, located in the central sections of the volume, and clearly visible without or with minimum overlap with other structures. In Fig 12A, average profiles of plasma and nuclear membrane points in local regions that are indicated in Fig 13 are shown, together with the three profile measures. Note that the horizontal axis on the profile figures indicates distances measured on the image from the membrane center, where negative values correspond to points at the inner side of cell/nuclear membranes. 3D surface models of the corresponding measured membrane points are also rendered in Fig 12B.

#### 3.2.3 Evaluation

Results of plasma and nuclear membrane segmentation from control and treated cells can be seen in Fig 13. It is worth noting from visual inspection that treated cells seem to have considerably larger nuclei compared to the total cell size, resulting in an apparent impression of “squeezing” the cytoplasm.

We observe that the area metric values of the nuclear membrane profiles in control cells are significantly higher than those of plasma membrane ones (Fig 12A), suggesting either a thicker membrane or membrane with more lipid concentration. This is partially consistent with the fact that under normal conditions, nuclear membranes are formed by a double lipid bilayer, as opposed to the single bilayer in plasma membranes. In contrast, in the treated cells, the area metric values of the nuclear membrane profiles are not remarkably larger than those of plasma membrane ones, suggesting a structural change compared to the known membrane composition in normal cells, or a change in the concentration of lipid.

The average membrane profiles measured on individual 2D slices are also studied, using points along the direction perpendicular to 2D membrane contours, an orientation similar to that of the three pixels involved for membrane thickness calculation in [6]. In Fig 12C, an example of average profiles for one slice in the control cell C1 are shown. The obtained profiles and feature values suggest that the profiles computed in 2D correlate very well with those obtained with the 3D approach, serving as a way to validate the profiles computed in 3D. However, the 3D average profiles present considerably less noise because profiles are computed using 3D membrane normals, which give more reliable information. Also, a larger number of membrane points are used.

Additionally, when comparing the membrane profile patterns, a few interesting observations seem to be worth mentioning. There seems to be a difference in grayscale values between the two sides of the immediate surroundings of the nuclear membranes: the outer sides, i.e. cytoplasm, in control cells have considerably higher grayscale intensities than the inner sides, while in treated cells the grayscale values do not seem to be significantly different on both sides. Ideally the contents enclosed by nuclear membranes are not infected by the peptide aggregation, thus we do not expect a difference inside nuclei between the control and treated cells. Therefore, what we observed from the membrane profiles could indicate a possible content concentration change in cytoplasm affected by the peptide treatment. Recalling the observations in Fig 13, this observation seems to be explainable by a denser absorbing content caused by the “squeezed cytoplasm” in treated cells. We thus speculate a possible abnormal cell arrangement probably induced by the alteration of cell membranes (permeability) by the peptide, which should be investigated further with larger amounts of data. This forms part of our future work.

## 4 Conclusion

The proposed 3D segmentation algorithm introduces the compatible ridge selection concept to better deal with noisy membrane structures. It has been validated on a synthetic image phantom using realistic microscope properties and structure dimensions, including the simulation of the missing wedge effects of cryo-TXM. Our segmentation approach constitutes minimal user effort with very satisfactory results, and we consider it only part of the contribution of the paper, that could eventually be changed by other method. Once properly segmented, a membrane quantification procedure is proposed through measuring features on membrane grayscale profiles perpendicular to the 3D membrane surfaces. These measures have been investigated using a synthetic membrane phantom that incorporates the PSF of the microscope. The newly introduced area metric has shown to be linearly related to the membrane thickness and the absorption coefficient of the membrane constituent material. This enables us to extract information about structural changes in membrane thickness or in the concentration of membrane components. Our segmentation and quantification algorithms require very little user interaction, thus reducing greatly the amount of time spent in manual analysis typically found in studies with data from soft X-ray transmission microscopes. The presented phantom validation, as well as real data experiments, have demonstrated the feasibility towards exploring the capability of cryo-TXM to detect changes in biological membranes at near native-state. Although we have shown theoretically that it is possible to obtain valid measures with a zone plate with a spatial resolution of 40*nm*, a better quantification with a zone plate with higher spatial resolution could be expected. The real data sets we have investigated are plasma and nuclear membranes of SH-SY5Y human neuroblastoma cells treated with Amyloid *β*-peptide to investigate its interaction with these membranes. From the results obtained with our method, we have validated our algorithms and showed that they work. Although results are preliminary, this pilot study has initiated a potential interesting biological question to be further investigated on larger numbers of datasets. We also expect that the methodology is not limited to the application addressed here, but could be applied to other type of cells and organelles to analyze their properties.

In conclusion, we have presented a 3D segmentation and quantification methodology for biological membranes of intact cells. We have achieved this on unfixed and unsectioned samples and revealed the 3D membrane morphology using synchrotron-based soft X-ray transmission microscopy. Our work is expected to help close the gap between the imaging of biological samples by electron and optical light microscopes. As synchrotron-based soft X-ray tomography continues to emerge worldwide, our work presents a useful tool of what the imaging field has to achieve: the routine analysis of high-resolution quantitative 3D structural information from cells in their native state. These studies are important for the better structural understanding of cells, and will lead to advances in bioimaging. Evaluation datasets from this work is publicly available at: https://zenodo.org/deposit/259703.
